# The Nurses’ Well-Being Index and Factors Influencing This Index among Nurses in Central China: A Cross-Sectional Study

**DOI:** 10.1371/journal.pone.0144414

**Published:** 2015-12-17

**Authors:** Runtang Meng, Yi Luo, Bing Liu, Ying Hu, Chuanhua Yu

**Affiliations:** 1 Department of Epidemiology and Biostatistics, School of Public Health, Wuhan University, Wuhan, Hubei, 430071, P. R. China; 2 School of Nursing, Ningbo College of Health Sciences, Ningbo, Zhejiang, 315100, P. R. China; 3 Center of Health Administration and Development Studies, Hubei University of Medicine, Shiyan, Hubei, 442000, P. R. China; 4 Global Health Institute, Wuhan University, Wuhan, Hubei, 430072, P. R. China; University of Helsinki, FINLAND

## Abstract

**Backgrounds/Objectives:**

A discussion and analysis of factors that contribute to nurses’ happiness index can be useful in developing effective interventions to improve nurses’ enthusiasm, sense of honor and pride and to improve the efficiency and quality of medical services.

**Methods:**

In this study, 206 registered nurses at the 2011 annual encounter for 12 Hanchuan hospitals completed a questionnaire survey that covered three aspects of the well-being index and thus served as a comprehensive well-being and general information tool.

**Results:**

Based on their index score, the nurses’ overall happiness level was moderate. The dimensions of the happiness index are listed in descending order of their contribution to the nurses’ comprehensive happiness levels: health concerns, friendly relationships, self-worth, altruism, vitality, positive emotions, personality development, life satisfaction and negative emotions. Four variables (positive emotion, life satisfaction, negative emotions, and friendly relationships) jointly explained 47.80% of the total variance of the happiness index; positive emotions had the greatest impact on the happiness index.

**Conclusions:**

Appropriate nursing interventions can improve nurses’ happiness index scores, thereby increasing nurses’ motivation and promoting the development of their nursing practice.

## Introduction

China is at the forefront of social concerns about medical work, and medical professionals have witnessed tremendous changes in their work environment in recent years. Studies have found that high levels of occupational stress can lead to feelings of self-doubt, irritability, and sleep disorders [1, 2]. Effort-reward, work-life imbalance, interpersonal conflict, general stress and burnout and can influence nurses’ subjective well-being [1–5]. Nurses are a special population because they experience high levels of stress in their everyday work [6, 7].

Happiness reflects the core indicators of subjective quality of life, the value that reflects a person’s happiness within a given period [8]. The nurses’ happiness index measures how happy nurses are with providing care; nurses with higher happiness index values have greater professional initiative [9, 10]. Employee performance-management features have different impacts on different aspects of well-being; and emotional demands from the nursing profession can act as challenges which promote motivation and well-being [11, 12].Therefore, the happiness index of nurses directly affects the efficiency and quality of medical services; consequently, understanding and improving nurses’ quality of life is important. In this paper, 206 registered nurses from 12 hospitals in Hanchuan completed a questionnaire survey. Based on the results of the survey, we analyzed the impact of factors that affect nurses’ happiness index values with the aim of using that information to develop effective interventions. The survey results are reported below.

## Methods

### Samples

From June to July 2011, 206 registered nurses from 12 hospitals of various levels in Hanchuan City, Hubei Province, were recruited using a random sampling method. A total of 220 questionnaires were sent out, and 160 questionnaires were distributed by nurses in three secondary hospitals (The People's Hospital, a Chinese medicine hospital, and a maternal and child health care hospital); 60 were distributed by nurses in nine primary care hospitals (8 township health centers and 1 community health service center). A total of 206 valid questionnaires were completed, and the questionnaire return rate was 93.64%. The subjects’ inclusion criteria were as follows: currently working, had obtained a medical practice certificate, and had no mental illness or disturbance of consciousness when the study was conducted. The exclusion criteria were as follows: re-employment after retirement, retired nurses and nurses who were engaged in advanced studies.

### Survey Tools

The general information questionnaire for nurses included hospital level (primary, secondary, or tertiary), age, length of service, work department, level of education, job title, employment status, marital status, and monthly income. The Multiple Happiness Questionnaire (MHQ) [13] included nine dimensions: life satisfaction(5 items), positive emotions (6 items), negative emotions(6 items), life vitality(6 items), health concern(5 items), altruism behavior(5 items), self-worth(5 items), friendly relationship(3 items) and personal growth(9 items). These dimensions were measured with 50 items using a Likert 7-level scale. For items A1-A38, the scale ranged from very uncomfortable to very comfortable, among them, entries A12 and A14, take the reverse scoring method; and for items B1-B12, the scale ranged from never to always; this study used a Likert 7 level score, and a reversed scale was used to score negative emotions. Higher scores indicated a stronger sense of happiness. A 9-level Likert scale was used to rate happiness (happiness index) from very unhappy to very happy: 1 indicated a low level of happiness, 3.67 indicated a moderate level of happiness, and 6.33 and higher indicated a high level of happiness. There are in this questionnaire, nine aspects belonging to psychological well-being and subjective well-being respectively. The 9 dimensions of the research on the MHQ were calculated respectively by Cronbach's coefficient, which is between 0.9056 and 0.6742, among them, the friendship between the highest (0.9056), the personality development dimension is lower (0.6742) [13]. In this study, the scale’s overall coefficient of internal consistency (Cronbach’s alpha value) was 0.941.

### Survey Methods and Ethics Statement

To ensure the validity and consistency of the questionnaire survey, the trained researchers engaged in conversation and communication with the management staff of the participating hospitals and health service centers; they then explained to the respondents how to complete the questionnaire, providing identical instructions to each participant. Each respondent was given ten to thirty minutes to complete the form independently. Anonymity was maintained, and the forms were recycled. All collected data is summarized in S1 Table.

The Institutional Review Board of Wuhan University School of Medicine, China, approved the study protocol in S2 Table. This study followed the Helsinki Convention’s norms and later modifications as well as the uniform requirements for manuscripts submitted to biomedical journals. This team ensured that the data collection process to fully respect and protect personal privacy. Fill out the instructions of the questionnaire also have instructions, respondents (nurses) to fill in the questionnaire, on behalf of their informed consent, and acknowledged our questionnaire information is not registered. Their written consent to participate in this topic research.

### Statistical Methods

We employed double parallel data entry with EpiData (version 3.1, Lauritsen JM & Bruus M, Odense, Denmark) and consistency testing using the Statistical Package for the Social Sciences software (version 18.0, SPSS, Inc., Chicago, IL, USA) to conduct a descriptive analysis. Pearson’s correlation analysis and multiple linear stepwise regression analysis were also employed. All tests were two-sided, and statistical significance was set at *p*<0.05.

## Results

### General Information

A total of 206 female nurses were included in this survey; among them, 57 (27.7%) were working in primary hospitals, and 149 (72.3%) were working in secondary hospitals. Other participant characteristics are shown in Table 1.

**Table 1:** Demographic characteristics of and general information on hospital nurses in Hanchuan City (n = 206).

Item	Number (n)	Percentage (%)
Hospital level		
Level one hospital	57	27.7
Level two hospital	149	72.3
Age (years)		
≤20	62	30.1
21–30	93	45.1
31–40	29	14.1
≥41	22	10.7
Work experience (years)		
<1	62	30.1
1–5	77	37.4
6–10	22	10.7
11–15	15	7.3
16–20	10	4.8
>20	20	9.7
Department		
Clinical	194	94.2
Administrative	12	5.8
Education background		
Associate’s degree, Some or no college, or Secondary school	168	81.6
Bachelor’s degree	38	18.4
Professional title		
Nurse	143	69.4
Senior nurse	28	13.6
Nurse supervisor	31	15.0
Co-chief nurse superintendent	4	2.0
Employment status		
Official	60	29.1
Contract or temporary	146	70.9
Income level (yuan)		
≤999	104	50.5
1000–1999	88	42.7
≥2000	14	6.8

### Comprehensive Sense of Happiness: Happiness Index Scores

The results showed that the nurses had a moderate level of happiness. As shown in Table 2, according to the happiness index, the dimensions of comprehensive happiness are as follows, in descending order of importance: attention to health, friendly relationships, self-value, altruistic behavior, life vitality, positive emotions and personal growth, life satisfaction, and negative emotions.

**Table 2:** The nurses’ comprehensive well-being scores for the happiness index (n = 206, $\bar{x}$±S, score).

Dimension	Number of items	The highest theoretical value	Actual score	Index value	Rank
Happiness index	1	9	6.05±1.50	Medium	-
Comprehensive well-being score	50	350	231.54±37.14	0.662	-
Life satisfaction	5	35	21.34±6.01	0.610	8
Positive emotions	6	42	28.44±7.52	0.677	6
Negative emotions	6	42	13.16±4.93	0.313	9
Life energy	6	42	28.50±7.18	0.679	5
Health concerns	5	35	28.97±5.27	0.828	1
Self-value	5	35	26.80±5.51	0.766	3
Friendly relationships	3	21	16.71±3.81	0.796	2
Altruistic behavior	5	35	25.80±6.03	0.737	4
Personal growth	9	63	41.84±10.49	0.664	7

Note: The index value refers to the ratio of practical and theoretical points

### The Correlation between the Happiness Index and the Dimensions of the MHQ

As table 3 shows, the Pearson correlation analysis results indicated that except for negative emotions, all other dimensions of the MHQ were positively correlated with the happiness index (all *p*<0.001). Lower negative emotion scores indicate a higher happiness index; the opposite relationship applies for the other eight dimensions of the MHQ.

**Table 3:** Correlation analysis for the happiness index and each dimension of the MHQ.

Dimension	R value	p value
Life satisfaction	0.504	0.000
Positive emotions	0.567	0.000
Negative emotions	-0.417	0.000
Life energy	0.358	0.000
Health concerns	0.304	0.000
Self-worth	0.420	0.000
Altruistic behavior	0.383	0.000
Friendly relationships	0.452	0.000
Personality growth	0.470	0.000

### Regression Analysis of the Relationship between the Happiness Index and Each Dimension of the MHQ

This study set the happiness index as the dependent variable and the nine dimensions of the MHQ as the independent variables. In a certain range, the random variable X (independent variable) is subject to normal distribution, and the random variable Y (dependent variable) is given. By tests of Normality, we can see that the happiness index (= Y) is subject to normal distribution; Kolmogorov-Smirnov P = 0.200>0.05. The dependent measure is normally distributed. Each individual observation is independent of each other. According to the standards α_in_≤0.05 and α_out_≥0.10, a multiple linear stepwise regression analysis of the relationship between the happiness index and each dimension of the MHQ was conducted. Table 4 shows that the well-being index was positively related with positive emotions, life satisfaction, and friendly relationships but was negatively correlated with negative emotions. As for the regression equation model, the multiple correlation coefficient was R = 0.692 and the determination coefficient was R^2^ = 0.478, which indicates that the above four factors can explain 47.80% of the happiness index total variance, and among them, positive emotions had the highest impact on the happiness index.

**Table 4:** Regression analysis for the happiness index and each dimension of the MHQ.

Dependent variable	Predictive variable	B value	β value	t value	p value	R^2^ value	F value	p value
Happiness	Positive emotions	0.428	0.358	6.140	0.000	0.478	46.102	0.000
	Life satisfaction	0.301	0.242	4.149	0.000			
	Negative emotions	-0.405	-0.222	-4.078	0.000			
	Friendly relationships	0.187	0.159	2.901	0.004			

### Regression Analysis of the Relationships between the Happiness Index and General Information

Using the happiness index as the dependent variable and general information as the independent variable according to standards α_in_≤0.05 and α_out_≥0.10, a multiple linear stepwise regression analysis between the happiness index and general information was conducted. Table 5 shows that the happiness index was positively correlated with the stress response and professional titles and negatively correlated with work pressure. Regarding the regression equation model, the multiple correlation coefficient was R = 0.267, and the determination coefficient was R^2^ = 0.071, which indicates that stress coping styles, professional titles and work pressure can explain 7.10% of the happiness index total variance and among these factors, stress coping styles have the greatest impact on happiness.

**Table 5:** Regression analysis for the happiness index and general information.

Dependent variable	Predictive variable	B value	β value	t value	p value	R^2^ value	F value	p value
General information	Ability to handle pressure	0.825	0.156	2.237	0.026	0.071	5.161	0.002
	Professional titles	0.319	0.174	2.528	0.012			
	Work pressure	-0.380	-0.140	-1.994	0.047			

## Discussion

Happiness or a sense of happiness is an advanced human psychological experience. As the ultimate goal and ideal of life, happiness has a unique meaning. All human acts are a pursuit of happiness and are influenced by people’s imagined models of happiness. Therefore, different cultural backgrounds, environments and faiths may lead to different understandings of and attitudes toward happiness. Personal outlooks on life, values, and the world guide people to consciously pursue happiness. Nurses are a special group of individuals who work under high pressure, and hospital administrators have always struggled to find ways to relieve nurses’ occupational stress and prevent job burnout. Humanized management ideas can be applied to both the recipients of services and the medical nursing staff who provide services. From the perspective of psychology, happiness research mainly refers to the happiness index. The introduction of the happiness index provides a new way of thinking about the development of nursing manpower capital [14] that can improve nurses’ well-being and help them approach their work with physical and mental pleasure. All of these results would have significant effects on the provision of high-quality nursing service. This study shows that happiness is a type of comprehensive subjective feeling, and nurses’ happiness is influenced by a variety of internal and external factors.

### Nurses Responses Indicate a Moderate Level of Happiness

This study found that the general happiness index of nurses is at a moderate level. This finding indicates that local nurses’ physical and psychological conditions are not ideal, as the scores for the nine dimensions of the MHQ show large data range.

Regarding comprehensive well-being, the lowest and second-lowest scoring dimensions were negative emotions and life satisfaction, respectively. Negative emotions are a tremendous challenge for nurses because of tension between doctors and patients; daily encounters with patients afflicted by disease; patients’ mental status; nurses’ educational levels; and the many different patient needs that require responses and care from the nurses. Additionally, changes in the patients themselves and their families evoke sadness, anger and other negative emotions [15], which can actively affect nurses’ emotional well-being and have a negative impact on their work. The quality of the relationships among the factors that contribute to nurses’ general well-being is very important; in fact, these relationships have a greater impact on nurses’ happiness than their salaries do [16]. The low scores in life satisfaction indicate that the nurses are not satisfied with their overall living conditions, and their life aspirations and needs are not well met. Low scores for life satisfaction may be associated with lower income levels, limited time for rest or entertainment, reduced time with family, and other personal factors.

The highest and second-highest scoring dimensions were health concerns and friendly relationships. The high scores for health concerns indicate that nurses are concerned about their health; they want to maintain a good life, and their concern about their own health may be related to their working conditions and occupational characteristics. Every day, nurses encounter large numbers of unwell people, and their resulting awareness of the importance of health hazards and diseases may lead to this strong sense of concern about their own health. Regarding the high scores for friendly relationships, nurses have relatively harmonious interpersonal relationships, and excellent interpersonal skills can improve morale, resulting in a collective spirit of cohesion [17] and a good atmosphere among the nursing team, which would emphasize the positive effects of nursing.

### Analysis of the Factors that Influence the Nurses’ Happiness Index

The WHO definition of health is as follows: “Health is a state of complete physical, mental and social adaptation and not merely the absence of disease and weakness”. Health care professionals’ physical and mental health provides the foundation for their ability to provide patient services, and nurses realize the value of a high quality of life. This approach to defining a good life has come to be called “subjective well-being” (SWB) [18]. SWB is an aspect of the comprehensive sense of happiness. SWB has some relevance because it can help people find happiness, provide optimal stimulation and positive social contact, and produce a social identity. For female nurses, as their social roles change and their range of available occupations expands, their life satisfaction and job satisfaction strengthens [9, 19]. Job satisfaction, to some extent, affects the happiness index.

#### Emotional factors

Emotional factors also affect nurses’ happiness index. SWB is a subjective experience, and objective factors do not directly affect subjective well-being; however, through positive emotions, personal growth and other subjective experiences, subjective well-being is influenced indirectly [12, 20]. This study found that nurses are prone to burnout [21] after working several years in the field. In Lebanon, burnout is particularly common and severe among working nurses; there is a significant correlation between burnout and nurses’ mental health [22]. "The health care light" and other historical societal factors do not look at the nursing profession accurately; nurses are not receiving the proper respect in work or life, and nurses receive less social support, which they can receive from all types of family units. In the eyes of the majority of patients and their families, physicians decide who is to be master over their health or survival. Nurses in health care often work in a passive, subordinate position associated with increased respect toward doctors; under such circumstances, nurses are treated with apathy and may even be manhandled or disrespected. As a result of high risk factors coupled with the serious shortage of domestic nurses, the ratio of nurses to beds is less than 0.4:1. The nursing workload, imminent burnout, tension between doctors and patients and other adverse psychological conditions contribute to an increase in negative emotions and decreased self-identity among nurses. Karimi’s research study demonstrate the importance of emotional intelligence and presenteeism effects on nurses’ well-being; and we should require more nursing training and development to be done in relation to emotional intelligence [23].

#### Support factors

Social support means a nurse is recognized by society, only if nurses are satisfied with their work and life and have harmonious interpersonal relationships, social support can really provide help on material or information to increase their happiness, satisfaction, sense of belonging and ability of handling emergency. Positive social role of support, can improve nurses’ overall emotional index. Especially strong support from work can reduce the incidence of job burnout and simultaneously inspire nurses to learn from each other than further enhance SWB experience. Life satisfaction depends on the family environment, marital relationships and other personal factors. Studies and surveys show that marriage can improve peoples’ happiness level; the subjective happiness levels of those who are married are higher than those who are unmarried, divorced, separated or widowed [24]. Lee found that when nurses leave their jobs because of burnout and interpersonal conflict, the resulting workload shifts require the remaining nurses to work excessive hours with increased psychological stress. These factors can negatively impact families, especially marital relationships, because the clinical front line is busy all day and nurses have few opportunities to communicate with their family and friends or to talk about their confusion and distress regarding their income or their satisfaction with the attention they receive from their family. There is a significant correlation between the emotional relationship between husband and wife and the happiness index [25]. Therefore, improvements in income satisfaction, improvements in marital relations, the effective and timely resolution of negative emotions, and continued good health are important for enhancing a nurse’s happiness index. Psychology research has found that good relationships and self-reported happiness are the most important determinants of the happiness index. Aristotle described people as “social animals” to emphasize the importance of human relationships; thus, a long-term intimate relationship is a main goal pursued by most people. Mutual appreciation among colleagues, mutual gratitude, mutual love and mutual support ease the psychological pressure of nurses to some extent and improve their relationships with others [26]. Harmonious interpersonal relationships among colleagues help individuals maintain a good general state of emotion and protect them under stress. Diener found that very happy people have rich and satisfying social relationships, and good social relationships are universally important to the human mood [27].

#### Stress factors

Nursing workers are always busy at clinical front line, rarely with family or friends to talk to their confusion and distress, when they are confronted with plight, competition and risk, their stress response is also an important factor to influence happiness, support cannot effectively ease the pressure on the impact of the SWB. In addition, high anxiety levels about workplace violence and certain types of work were associated with experiences of violence; interventions to minimize workloads and improve nurse-patient relationships are essential to combat depressive symptoms among nurses [28, 29]. Violence, especially in the medical workplace, can cause direct physical or psychological harm to nurses, create a violent shadow victim mentality, and lead to considerable psychological pressure [30, 31], loss of motivation, and fewer happy experiences.

#### Professional titles factors

Professional titles are closely related with and material benefits, occupational status and career achievements. Nurses with high titles are mostly the backbone of the department or hospital and have a good self-control and sound social adaptability, namely, the higher the professional titles, the higher happiness index. Unit leadership that creates empowering workplace conditions plays a key role in establishing supportive practice environments that increase work effective-ness and improves well-being [32]. But for primary nurses, they get a slow promotion for a variety of reasons, so if there are more opportunities and platforms being offered, it will certainly help to improve the nurses’ happiness.

#### Other factors

Many other factors are affecting nurses’ happiness index as well, life vitality, self-value, altruistic behavior, personality growth to name only a few. Nursing job reflects their value of life, their passionate vitality of life, selfless dedication and a sound personality growth which all involves love, and that love propels nurses to experience happiness when they take care of patients.

In addition, a lot of research and studies have shown that empathy and comfort can effectively relieve fatigue and pain, improve nurses' well-being, and let nursing workers to work better [33–35]. For those nurses who are working on the frontline of clinical medicine, their happiness index is an important problem drawing attention from all walks of life. The asset-based paradigms of positive psychology offer new approaches for bolstering psychological resilience and promoting mental health [36]. Health administrators and policy-makers would like to enhance the work related to positive emotional experience by coping strategy and proper intervention, especially from the perspective of positive psychology.

The sample size of 206 respondents from twelve hospitals in one city may limit the study’s power. While this sample was adequate for our analysis, it is insufficient to allow for a more detailed analysis of differences in the workplace and happiness indexes across departments in different area hospitals. This cross-sectional study is limited to a small city in central China because of a lack of resources and time.

## Conclusion

In short, subjective well-being is a positive feeling and level of psychological awareness that is related to nurses’ mental health. If nurses’ subjective well-being can be effectively improved, burnout among nurses may be alleviated. Health service managers should measure and understand nurses’ happiness, consider the factors that affect nurses’ happiness, and provide active psychological counseling and care to promote nurses’ enthusiasm and inspire their dedication. Doing so would help nurses achieve true job satisfaction and promote their loyalty to their workplace.

## Supporting Information

S1 Table Data of questionnaire entry. Survey database.
(RAR)

S2 Table Ethical approval. Ethical approval was given by the Medical Ethics Committee of Wuhan University School of Medicine, P.R. China.
(PDF)

S3 AppendixThis file includes the following Tables 1, 2, 3, 4 and 5.Demographic characteristics of and general information on hospital nurses in Hanchuan City (n = 206).The nurses’ comprehensive well-being scores for the happiness index (n = 206, $\bar{x}$±S, score). Correlation analysis for the happiness index and each dimension of the MHQ. Regression analysis for the happiness index and each dimension of the MHQ. Regression analysis for the happiness index and general information.(RAR)
