# Supplementary material for: The Nurses’ Well-Being Index and Factors Influencing This Index among Nurses in Central China: A Cross-Sectional Study
Source: PLoS One. 2015 Dec 17;10(12):e0144414. doi: 10.1371/journal.pone.0144414 (PMC4682979; doi:10.1371/journal.pone.0144414)
Supplement: S2 Table — Ethical approval was given by the Medical Ethics Committee of Wuhan University School of Medicine, P.R. China. (PDF) [file pone.0144414.s002.pdf]

武汉大学医学部伦理委员会审批件

编号：

研究项目名称	湖北汉川基层医务人员工作价值观和幸福感调查		
研究内容和意义	随着生命科学和医学科学技术的日益发展及医疗模式的不断转变，在临床实践过程中，医务人员的传统价值观和幸福感受到极大的冲击，面对着越来越多的潜在的价值冲突如多种伦理原则的相互冲突、个人的道德准则与特定的职业伦理要求间的相互冲突。目前对医务人员专业价值观和幸福感的研究也是近年来西方国家研究的一个热点，国内对这一问题也开始关注。然而，国内针对基层医疗机构医务人员幸福感和工作价值观的研究还较少，关注基层医疗机构医务人员工作价值观和幸福感，对认识与改善其健康水平和生活质量具有积极的指导意义。本研究采用综合幸福感问卷（MHQ）和采用 Elizur 等编制的工作价值观量表，运用便利抽样法调查湖北汉川医疗机构医务人员。<br>每个入选的医生和护士将要求填写一份调查问卷，此次调查采用完全匿名的方式。调查问卷中不会收集医生护士的姓名和其他可用于个人识别的相关信息，也不会从医疗机构管理者处收集信息，本课题组确保数据收集过程中对个人隐私权的充分尊重和保护。		
申请单位	公共卫生学院	项目负责人	孟润堂

医学伦理委员会审批意见：

经审核符合相关规定，同意开展该项目。

[illegible] [illegible] [illegible] [illegible] [illegible]

刘晴

武汉大学医学部伦理委员会

2011 年 6 月 10 日

（印章：武汉大学医学部 医学伦理委员会）
